# Molecular circadian rhythm shift due to bright light exposure before bedtime is related to subthreshold bipolarity

**DOI:** 10.1038/srep31846

**Published:** 2016-08-22

**Authors:** Chul-Hyun Cho, Joung-Ho Moon, Ho-Kyoung Yoon, Seung-Gul Kang, Dongho Geum, Gi-Hoon Son, Jong-Min Lim, Leen Kim, Eun-Il Lee, Heon-Jeong Lee

**Affiliations:** 1 Department of Psychiatry, Korea University College of Medicine, Seoul, South Korea; 2 Sleep-Wake Disorders Center, Korea University Anam Hospital, Seoul, South Korea; 3 Department of Biomedical Science, Korea University College of Medicine, Seoul, South Korea; 4 Department of Psychiatry, Gachon University School of Medicine, Incheon, South Korea; 5 Department of Legal Medicine, Korea University College of Medicine, Seoul, South Korea; 6 Department of Lighting Environment Research, Korea Institute of Lighting Technology, Seoul, South Korea; 7 Department of Preventive Medicine, Korea University College of Medicine, Seoul, South Korea

## Abstract

This study examined the link between circadian rhythm changes due to bright light exposure and subthreshold bipolarity. Molecular circadian rhythms, polysomnography, and actigraphy data were studied in 25 young, healthy male subjects, divided into high and low mood disorder questionnaire (MDQ) score groups. During the first 2 days of the study, the subjects were exposed to daily-living light (150 lux) for 4 hours before bedtime. Saliva and buccal cells were collected 5 times a day for 2 consecutive days. During the subsequent 5 days, the subjects were exposed to bright light (1,000 lux), and saliva and buccal cell samples were collected in the same way. Molecular circadian rhythms were analyzed using sine regression. Circadian rhythms of cortisol (F = 16.956, p < 0.001) and relative *PER1/ARNTL* gene expression (F = 122.1, p < 0.001) showed a delayed acrophase in both groups after bright light exposure. The high MDQ score group showed a significant delay in acrophase compared to the low MDQ score group only in salivary cortisol (F = 8.528, p = 0.008). The high MDQ score group showed hypersensitivity in cortisol rhythm shift after bright light exposure, suggesting characteristic molecular circadian rhythm changes in the high MDQ score group may be related to biological processes downstream from core circadian clock gene expression.

Bipolar disorder (BD) is a chronic mental illness characterized by alternations in mood, activity, sleep, and energy. Patients with BD suffer from marked functional impairments and a reduced quality of life^1^. As a result, BD is considered to be a major social burden^2^. In order to ameliorate this social burden, it is important to develop methods for the accurate diagnosis of BD, to prevent its onset, to provide more appropriate therapeutic strategies for its treatment, to increase the duration of inter-episode periods, and to improve its prognosis.

Research on the association between BD and circadian rhythms is an important key to understanding BD. Disturbances in circadian rhythms have been suggested to be core features of BD and are thought to play a fundamental role in BD pathophysiology^3^. A number of studies conclude that disruptions^4^,^5^, phase shift^6^,^7^, or instability^8^ of circadian rhythms are related to BD. Moreover, genetic studies have suggested an association between circadian genes and BD^9^,^10^,^11^,^12^. The therapeutic effects of mood stabilizers such as lithium or valproate in treating BD are believed to be due to effects on the rhythmic properties of circadian genes and molecular clocks via the inhibition of glycogen synthase kinase-3β^13^,^14^.

Light plays a primary role in the regulation of circadian rhythms, and light exposure is the main environmental cue for the synchronization of circadian rhythms in humans^15^. Excessive exposure to light at inappropriate times or a lack of light exposure at the required time may be involved in the pathophysiology of some mood disorders. Controlled therapeutic light exposure is usually used to treat certain mood disorders^15^. A previous study concluded that some subjects may be more sensitive to variations in natural bright light and that an increased sensitivity to light may be related to the development of affective disorders^16^. Seasonal variations in manic episodes have been reported to be related to the number of hours of daily sunshine^17^. Therefore, sensitivity to light exposure may be a characteristic feature of mood disorders.

A number of studies have investigated a broad range of variables, such as genetic factors, phenotypic manifestations, biomarkers, and neuroimaging patterns, in normal subjects at high risk for developing BD^18^. There are various methods used for the selection of subjects at risk of developing BD, including familial history of BD^19^ or clinical features such as early onset and severity of depression, recurrent and atypical depression, subclinical mania, and psychosis^20^. Another method for selecting at-risk subjects is the use of clinical scales such as the childhood bipolar questionnaire^21^, the bipolar-at-risk criteria^22^, or the mood disorder questionnaire (MDQ)^23^.

We hypothesized that normal subjects with high MDQ scores would be more sensitive to bright light exposure before bedtime and thus show more dramatic changes in molecular circadian rhythms, sleep parameters, and behavioral rhythms compared to subjects with low MDQ scores. We thus designed and performed a controlled experimental study to investigate this potential characteristic marker of subthreshold bipolarity in normal subjects.

## Results

### Circadian rhythm of cortisol concentration in saliva

**Figure 1:**
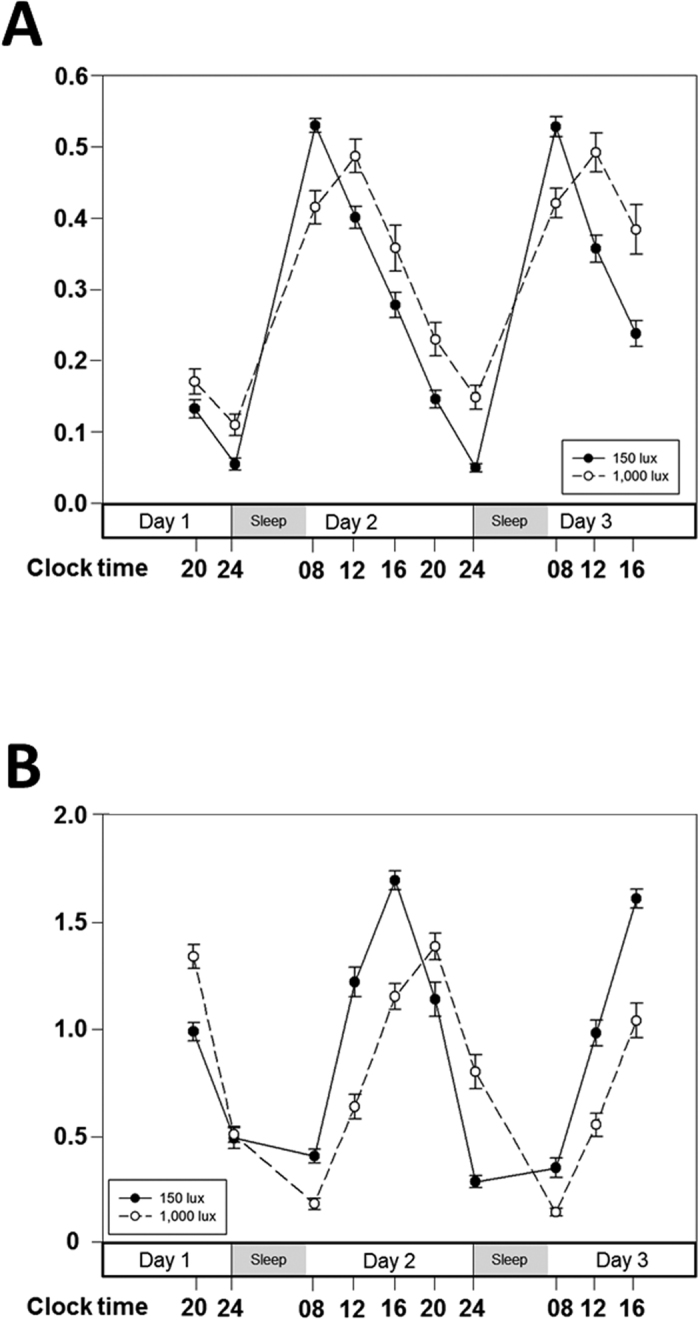
Altered molecular circadian rhythms of salivary cortisol and relative *PER1/ARNTL* expression levels following bright light exposure before bedtime. Twenty-five young, healthy male subjects were exposed to 150 lux artificial light at night (ALAN) before bedtime for 2 consecutive days. The circadian rhythms of salivary cortisol concentration (**A**) and relative *PER1/ARNTL* expression levels (**B**) are indicated as black circles connected with a continuous black line. After the 150 lux ALAN exposure, the same 25 subjects were exposed to 1,000 lux ALAN for 5 consecutive nights. The circadian rhythms of salivary cortisol concentration (**A**) and relative *PER1/ARNTL* expression levels (**B**) are indicated by white closed circles connected with a dashed black line. Data are expressed as mean ± standard error of the mean (SEM).

To investigate the impact of bright artificial light at night (ALAN) exposure before bedtime on circadian rhythm of cortisol concentration, we analyzed salivary cortisol concentrations of all 25 subjects using paired *t*-tests (Table 1 and Fig. 1). A significantly delayed acrophase in salivary cortisol concentration (t = −3.927, p = 0.001) was found in subjects after bright ALAN exposure. The mesor of cortisol concentration also significantly increased between nights (t = −2.494, p = 0.02). We found that bright ALAN exposure before bedtime in healthy adults significantly delayed the acrophase and increased the mesor of the circadian rhythm of cortisol concentration.

**Figure 2:**
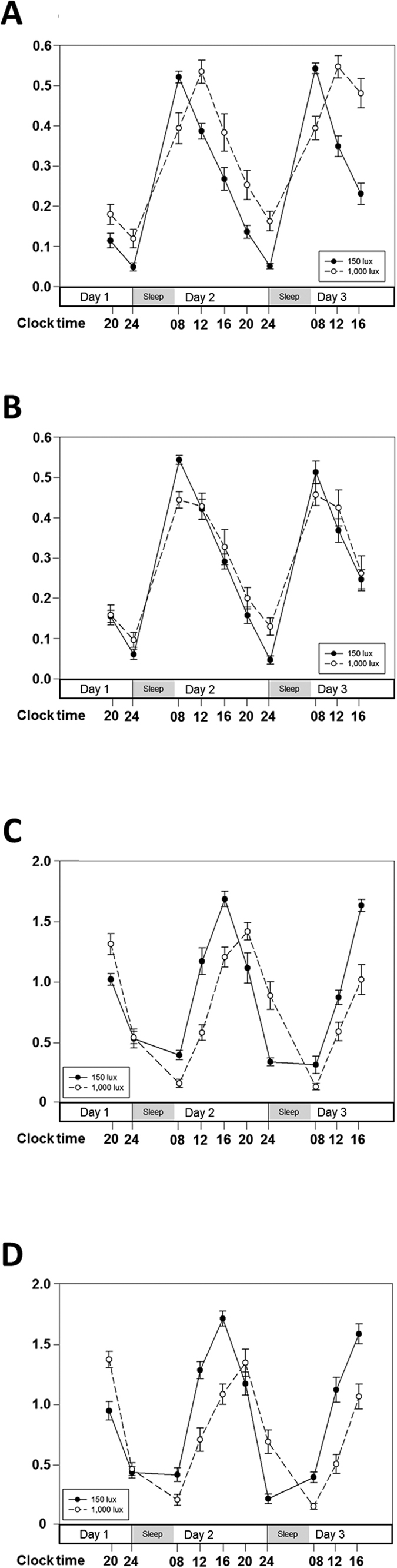
Altered molecular circadian rhythms of salivary cortisol concentration and relative *PER1/ARNTL* expression levels following bright light exposure before bedtime in high and low mood disorder questionnaire (MDQ) score groups. Fourteen young, healthy male subjects with subthreshold bipolarity (high MDQ score group) were exposed to 150 lux artificial light at night (ALAN) for 2 consecutive days. The circadian rhythms of salivary cortisol concentration (**A**) and relative *PER1/ARNTL* expression levels (**C**) are indicated by black circles connected with a continuous black line. After exposure to 150 lux ALAN, the same 14 subjects in the high MDQ score group were exposed to 1,000 lux ALAN for 5 consecutive days. The circadian rhythms of salivary cortisol concentration (**A**) and relative *PER1/ARNTL* expression levels (**C**) are indicated by white closed circles connected with a dashed black line. Eleven young, healthy male subjects with low risk of bipolar disorder (low MDQ score group) were exposed to 150 lux ALAN for 2 consecutive days. The circadian rhythms of salivary cortisol concentration (**B**) and relative *PER1/ARNTL* expression levels (**D**) are indicated by black circles connected with a continuous black line. After exposure to 150 lux ALAN, the same 11 subjects in the low MDQ score group were exposed to 1,000 lux ALAN for 5 consecutive days. The circadian rhythms of salivary cortisol concentration (**B**) and relative *PER1/ARNTL* expression levels (**D**) are indicated by white closed circles connected with a dashed black line. Data are expressed as mean ± standard error of the mean (SEM).

Next, we analyzed the changes in circadian rhythm of cortisol concentration over the course of the study between the two groups, namely the high MDQ score group and the low MDQ score group, after bright ALAN exposure using a repeated measures ANOVA (Table 2 and Fig. 2). Both groups showed a significantly delayed acrophase (F = 16.956, p < 0.001) and a significant increase in mesor (F = 5.678, p = 0.026) of salivary cortisol concentration over the course of the study. The most noteworthy finding of the present study is the night—group interaction observed in the acrophase of the salivary cortisol concentration. We observed that the acrophase in the high MDQ score group was significantly delayed compared with that in the low MDQ score group (F = 8.528, p = 0.008). Altogether, bright ALAN exposure before bedtime caused a significantly delayed acrophase in cortisol concentrations in both groups. Moreover, the high MDQ score group was more sensitive to bright ALAN exposure, showing a significant increase in acrophase delay in cortisol concentration compared with the low MDQ score group.

### Circadian rhythm of relative *PER1/ARNTL* gene expression levels in buccal epithelial cells

To study the impact of bright ALAN exposure before bedtime on circadian rhythm of relative *PER1/ARNTL* gene expression, we used the same analysis as performed on cortisol concentration (Table 1 and Fig. 1). We found a significant delay in acrophase (t = −11.146, p < 0.001) and a significant decrease in mesor of the relative *PER1/ARNTL* gene expression ratio (t = 2.638, p = 0.014) in subjects after bright ALAN exposure. The amplitude of relative *PER1/ARNTL* gene expression levels was significantly suppressed between nights after bright ALAN exposure (t = 6.555, p < 0.001). We confirmed that bright ALAN exposure before bedtime in healthy adults significantly delayed the acrophase, decreased the mesor, and suppressed the amplitude of the circadian rhythm of the relative *PER1/ARNTL* gene expression ratio.

We investigated the changes in circadian rhythm of relative *PER1/ARNTL* gene expression ratio over the course of the study in the two groups after bright ALAN exposure using a repeated measures ANOVA (Table 2 and Fig. 2). Both groups exhibited a significantly delayed acrophase (F = 122.1, p < 0.001), a reduced amplitude (F = 44.774, p < 0.001), and a decrease in mesor (F = 7.338, p = 0.013) of relative *PER1/ARNTL* gene expression levels during the course of the study. However, there was no night—group interaction of the *PER1/ARNTL* gene expression ratio and no difference between groups.

### Behavioral rhythm of actigraphy data

There was no significant change between nights in the behavioral rhythm of actigraphy data after bright ALAN exposure before bedtime in subjects as a whole (Table 3). A repeated measures ANOVA on the behavioral rhythm showed no statistically significant differences between nights of the study in both groups. However, we found significant night—group interactions for the period of the behavioral rhythm (F = 7.844, p = 0.01), as shown in Table 4. The period of the behavioral rhythm in the high MDQ score group increased, whereas that in the low MDQ score group decreased between nights.

### Sleep parameters of polysomnography

We analyzed sleep parameters of polysomnography in 21 subjects using the same method as above and found no significant differences between nights in subjects as a whole (Table 3). As shown in Table 4, a repeated measures ANOVA on sleep parameters showed no statistically significant differences between nights in both groups. However, there were significant night—group interactions for sleep parameters in stage N3 (F = 4.785, p = 0.041) and TA (F = 5.0, p = 0.038). Stage N3 showed a decreasing tendency in the high MDQ score group and an increasing tendency in the low MDQ score group. Stage TA showed an increasing tendency in the high MDQ score group and a decreasing tendency in the low MDQ score group.

## Discussion

We performed a controlled experimental study on young, healthy male adults divided into high and low MDQ score groups to investigate whether bright ALAN exposure affects molecular circadian rhythms, sleep parameters, and behavioral rhythms.

Both circadian gene rhythms and hormonal rhythms were shown to have a significant delay of acrophase after bright ALAN exposure when groups were considered separately and when all subjects were grouped together. Almost 99% of the populations of the U.S. and European countries experience significant ALAN. As a result, exposure to ALAN has become a part of modern everyday life^24^,^25^,^26^. Various studies have reported negative impacts of ALAN exposure on humans^15^,^25^,^27^. Bright light is a powerful factor in resetting the human circadian pacemaker independent of the timing of the sleep-wake cycle^28^,^29^. Therefore, bright light exposure may have broad effects on humans resulting from disturbance of the circadian system. Research on the effects of bright ALAN exposure on humans is limited^30^,^31^. Our study provides evidence that bright ALAN exposure just before bedtime significantly changes molecular circadian rhythms. Since circadian rhythms are closely related to a wide range of endocrine^32^, immune^33^, physical^34^, and mental^35^,^36^ states, it is very important to limit bright ALAN exposure before bedtime.

The most noteworthy finding of the present study is the observation of significant molecular circadian rhythm changes after bright ALAN exposure between the two different groups over the course of the study. Specifically, we observed a significantly delayed acrophase in the cortisol circadian rhythm after bright ALAN exposure in the high MDQ score group when compared to the low MDQ score group. This remarkable finding leads us to suggest that the hypersensitivity of the cortisol circadian rhythm to bright light exposure may be used as a possible biological marker for determining the risk of BD. Cortisol, an adrenal hormone essential for lipid and glucose metabolism, has been used as an important tool for studying circadian rhythms^37^,^38^,^39^,^40^ as its circadian rhythm is synchronized with light exposure^41^. Because cortisol and its circadian rhythms are closely related to various mental states such as stress^42^, major depression^43^,^44^, post-traumatic stress disorder^45^,^46^, and BD^47^,^48^, it is important to study the relationship between the circadian rhythm of cortisol and the risk of BD. Interestingly, studies on plasma melatonin levels suggest that hypersensitivity to light may be a possible marker for manic-depressive illness^49^,^50^. Numberger *et al.* reported a hypersensitivity to melatonin suppression by light in young, healthy people at high risk for major affective disorders^51^. Karen *et al.* reported that melatonin secretion and sensitivity to bright nocturnal light are highly heritable^52^. The present study on multi-dimensional variables including cortisol levels is especially meaningful, as research on cortisol has been somewhat lacking in comparison to research focused on melatonin^48^,^53^. Several studies have examined cortisol in unaffected offspring of parents with BD, revealing a putative relationship to BD risk^54^,^55^. However, these studies provide limited information because cortisol levels were checked not by its circadian rhythm but by its simple level for measurement. To further investigate the link between cortisol levels and BD risk, the present study measured and analyzed the changes in the circadian rhythm of cortisol after bright ALAN exposure.

In addition to the circadian rhythms of cortisol concentration in saliva, this study also observed circadian rhythms of gene expression in buccal mucosa. The regulation of circadian gene expression may reflect the circadian systems of peripheral cells as well as the influence of cortisol rhythms and melatonin reflecting a central pacemaker localized in the suprachiasmatic nucleus (SCN)^56^. Several previous studies have shown that circadian rhythms of circadian gene expression occur not only in the SCN but also in peripheral organs^57^,^58^,^59^,^60^,^61^. In this study, to observe circadian rhythms of gene expression, we used mRNA extracted from buccal epithelial cells. In the preliminary research of this study, we tested five circadian genes (*ARNTL*, *PER1*, *PER2*, *PER3*, and *NR1D1*) extracted from buccal epithelial cells of five healthy people (independent from the present main study) who had been confirmed to show regular circadian rhythms by actigraphy and salivary cortisol concentration. However, the preliminary result did not show distinct circadian rhythms of gene expressions when each gene was observed alone (Supplementary Figure S1A). It was because of several characteristics of buccal epithelial cells. First, buccal epithelial cells are necessarily collected together with saliva, which destroys RNA to protect the host from viral infection. Second, it is impossible to obtain the same number of buccal epithelial cells in each collection (performed by gently scraping the inner cheek using a cytological brush), leading to varying amounts of mRNA. Therefore, we decided to revise mRNA expression values by calculating their expression by ratio, as performed and validated in a previous study^62^. Among observed mRNA expression of five circadian genes, we observed more relevant circadian rhythms of *ARNTL* and *PER1* than in the others. The circadian rhythms of *ARNTL* and *PER1* were inverse in phase to each other (Supplementary Figures S1A and S2), in agreement with previous studies^12^,^62^,^63^,^64^,^65^. Accordingly, to obtain the most distinguishable circadian rhythms, we investigated the relative gene expression of *ARNTL* and *PER1* and showed that the ratio of *PER1*/*ARNTL* in buccal epithelial cells is a reliable method for measuring circadian rhythms of peripheral circadian gene expression (Supplementary Figures S1B and S2). Circadian gene expression patterns are known to directly reflect circadian rhythms in humans^66^. For this reason, the *PER1/ARNTL* gene expression ratio is thought to show the effects of bright ALAN exposure more directly and sensitively than other measures. However, we observed that the cortisol circadian rhythm was much more delayed than the *PER1*/*ARNTL* expression rhythm in the high MDQ score group compared to the low MDQ score group. We believe that this difference between the cortisol rhythm and the circadian gene rhythm suggests that BD risk is not related to circadian genes themselves but to the regulation of the circadian rhythm process after changes in the expression of core circadian genes.

Gaspar *et al.* reported that human genetic differences in major signaling pathways may be related to the light-dependent suppression of melatonin in BD^67^. Cortisol expression, which exhibits circadian rhythms reminiscent of those of melatonin, may be differentially affected by genetic differences in signaling pathways related to light-dependent changes based on the MDQ scores of subjects. On the other hand, expression levels of circadian genes are not considered suitable markers for predicting subthreshold bipolarity, as these genes are more directly affected by and more sensitive to bright ALAN exposure. The changes in these markers may thus predominantly reflect the effects of bright ALAN exposure.

We found significant night—group interactions for stage N3 and TA sleep parameters, as well as for the period of the behavioral rhythm. The night—group interactions for stage N3 and TA suggest that subjects with high MDQ scores may be more negatively affected by bright ALAN exposure before bedtime resulting in decreased deep sleep and increased arousal during sleep. However, interpretation of the results from this study is limited by the fact that parameters of stage N3 and TA and the period of the behavioral rhythm did not show significant differences between nights in either group.

We aimed to investigate the impact of bright ALAN exposure on a number of genetic, hormonal, and behavioral measures in normal subjects with high MDQ scores. The acrophase of the cortisol circadian rhythm was the only variable found to predict subthreshold bipolarity. This finding suggests that there may be a critical step related to BD along the pathway from gene expression to hormone production. This study was limited by a relatively small number of subjects and the fact that we were not able to specifically identify the pathway that determines BD risk. However, this study provides a significant advance in the field because it describes an experimental approach to examine our hypothesis, thereby introducing a meaningful way to elucidate causality of BD onset and recurrence in addition to identifying a putative method for predicting the risk of BD in normal subjects. In the future, we hope to verify the results of the present study and to advance our understanding further by using intensive molecular techniques on a larger number of subjects.

## Methods

### Subjects

From September 2013 to August 2014, a total of 29 healthy male adults ranging from 20 to 30 years of age (mean ± SD: 26.00 ± 2.89) were recruited for this study. For purposes of homogeneity, we decided to use only male subjects because data from female subjects may be more strongly influenced by hormonal changes such as the menstrual cycle. Advertisements for volunteers who “sleep like a baby” and “keep regular sleep-wake cycles” were posted on the internet bulletin boards of the Korea University to recruit these subjects. All volunteers were interviewed by psychiatrists specializing in sleep (authors HJL and CHC) in order to exclude volunteers who were overweight and suspected of snoring. Through in-depth interviews by psychiatrists with all volunteers, we confirmed that subjects had no personal or familial psychiatric history. All participants completed questionnaires regarding their sleep conditions and physical and psychiatric health. In particular, we gathered information about typical sleep patterns during weekdays and weekends as well as sleep environment and hygiene, including patterns of caffeine, alcohol, cigarette, and drug use. The MDQ, a subjective self-report screening tool, was used to assess subthreshold bipolarity. A high MDQ score (usually 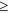
7) is associated with an increased probability of BD^23^. The MDQ has been used as an assessment tool not only for the study of BD^23^,^68^ but also for study of the risk of BD in the general population^69^,^70^ or in subjects with major depressive disorder^71^,^72^. All participants provided informed written consent prior to enrollment after a full explanation and understanding of this study. The study protocol was approved by the Institutional Review Board of Korea University Anam Hospital (AN12261-010) and was conducted in accordance with the Declaration of Helsinki.

During the week prior to the main experiment, all participants were asked to wear a wrist actigraph (Actiwatch-L^®^, Mini Mitter) to verify that they were keeping a regular sleep-wake cycle. After reviewing the actigraphy data for each participant, we found one participant who showed a disturbed sleep-wake cycle characterized by excessive napping. Three participants dropped out of the study for personal reasons. The remaining 25 participants completed the entire experimental process. These participants were divided into two groups based on their MDQ mood scores: the high MDQ score group (MDQ score ≥ 7; mean ± SD: 9.571 ± 1.697; 14 subjects), and the low MDQ score group (MDQ score < 7; mean ± SD: 2.091 ± 2.212; 11 subjects). Student’s *t*-test or the Mann-Whitney test was performed to compare demographic characteristics between the high and low MDQ score groups according to a normality assumption. There were no differences in age (mean ± SD: 25.64 ± 2.53 vs. 24.91 ± 1.81, p = 0.43), weight (kg; 68.31 ± 10.43 vs. 70.10 ± 4.11, p = 0.60), or height (cm; 174.07 ± 5.55 vs. 176.45 ± 5.65, p = 0.30) as determined by Student’s *t*-tests between high and low MDQ score groups, respectively. Similarly, there were no differences in education between the two groups (p = 0.168) as determined by a Mann-Whitney test.

### Protocol

**Figure 3:**
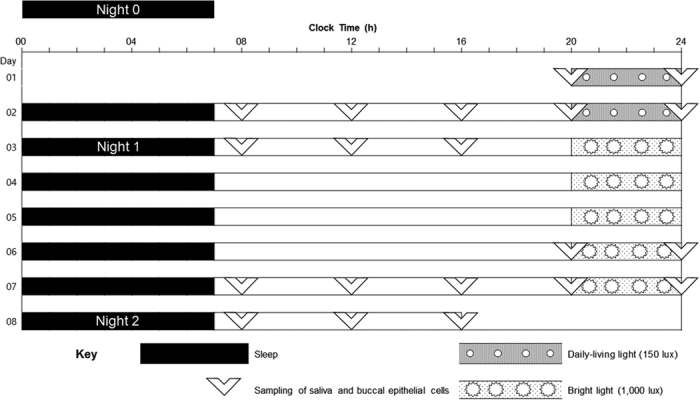
Protocol design. Sleep was scheduled from 00:00 to 07:00 during the 8 days of the experiment. On study days 1 and 2, the participants were exposed to 150 lux daily-living artificial light at night (ALAN) before bedtime. On study days 3, 4, 5, 6, and 7, the participants were exposed to 1,000 lux bright ALAN before bedtime. All daily-living and bright ALAN exposures were scheduled from 20:00 to 24:00. Samplings of salivary and buccal epithelial cells were scheduled with 4-hour intervals starting at 20:00 on study day 1 to 16:00 on study day 3 and from 20:00 on study day 6 to 16:00 on study day 8. No samples were collected during sleep times. Nocturnal polysomnography (NPSG) was conducted at three time points (Night 0, Night 1, and Night 2). Night 0 was in fact 3 nights before the main experimental study, during the regular sleep-wake cycle and daily-living ALAN exposure before sleep in order to reduce interference from the first-night effect. Nights 1 and 2 were the last nights of the 150 lux and the 1,000 lux ALAN exposure, respectively.

Figure 3 shows an overview of the protocol used in this study. Beginning at one week prior to the main experiment, all participants were prohibited from napping during the daytime and permitted to sleep only at night in order to keep regular sleep-wake cycles. Sleep logs and wrist actigraph records were used to monitor adherence to these guidelines. All participants were prohibited from consuming medicine, coffee, or alcohol, because of potential effects on sleep-wake regularity. Moreover, they were instructed to limit light exposure to daily-living light after 20:00 h and were required to sleep at midnight and to wake up at 07:00 h. All participants maintained their normal daily living and slept in their own beds at home.

The main experimental study was carried out for 8 consecutive days and 7 nights. The study was divided into two phases based on differences in the intensity of ALAN: 2 nights at 150 lux and 5 nights at 1,000 lux. We designed and installed a controllable light box system in the experimental room to create the differences in light exposure. The system was installed by an illumination expert affiliated with the Korea Institute of Lighting Technology. The light box was installed in the ceiling of the experimental room so that the light illuminated the whole room. The experimental room was 30 m^2^ in size and had seven light-emitting diode (LED) lights, each with a size of 120 cm × 30 m, evenly distributed along the ceiling. The light illuminated the entire room and the light intensity was controlled using a dimmer. Only the staff were permitted to use the dimmer and control the intensity of lighting; all participants were prohibited from using the dimmer.

The subjects maintained their usual daily routine activities outside the unit during the daytime while wearing wrist actigraphs for monitoring their daily routine. Subjects were exposed to daily-living light (150 lux) for 4 hours (from 20:00 h to 24:00 h) before bedtime during the first 2 consecutive nights of the study. They were then exposed to bright light (1,000 lux) for 4 hours (from 20:00 h to 24:00 h) before bedtime during the next 5 consecutive nights of the study. The light intensity in the experimental room was checked on a nightly basis by an illuminometer (ANA-F11, Tokyo Photo, Japan) placed at horizontal eye level when sitting upright in a chair. Subjects read books or studied independently but were prohibited from watching television or using smartphones or tablets in the experimental room. All participants went to bed at the clinical trial center in the Korea University Anam Hospital every night of the study period, except during the nocturnal polysomnography (NPSG) session, which was performed in the sleep lab of the Sleep-Wake Disorders Center in the Korea University Anam Hospital. They were required to sleep at midnight and to wake up at 07:00 h during the study period in order to keep a consistent time-in-bed.

Saliva and buccal epithelial cells were collected from the subjects in order to analyze the circadian rhythms of cortisol and circadian gene expression. Sample collection was performed at 08:00 h, 12:00 h, 16:00 h, 20:00 h, and 24:00 h for the last 2 consecutive days of the experiment and was carried out by trained staff in the monitoring unit of the Sleep-Wake Disorders Center. The subjects were instructed to always be in the monitoring unit on time at every sampling point, so subjects maintained their daily routines during the 2 consecutive sampling days in the monitoring room. The subjects provided saliva samples directly into Salivettes (Sarstedt AG & Co., Nümbrecht, Germany) at each of the specified time points. The Salivette device was used according to the manufacturer’s instructions and stored at −20 °C until the time of the assay. Immediately after saliva sample collection, buccal epithelial cell samples were collected by gently scraping both inner cheeks using a cytological brush. The buccal epithelial cell samples were immediately placed into RNAlater reagent (Sigma-Aldrich, St. Louis, MO, USA) and were then maintained at −20 °C until the time of analysis. The saliva samples were used to for the cortisol assay, and each participant’s cortisol circadian rhythm was determined. cDNA was synthesized from total RNA of collected buccal epithelial cells, and circadian gene expression rhythms were determined using the *PER1/ARNTL* expression ratio.

NPSG was conducted on 3 different nights during the study period. Three nights before the main experiment, each participant underwent an initial NPSG session (Night 0) while maintaining a regular sleep-wake cycle and normal ALAN exposure before bedtime to reduce interference from the first-night effect. The second and third NPSG sessions (Nights 1 and 2) were conducted on the last night of each ALAN schedule.

### Measurements

#### Measurement of cortisol concentration in saliva

The Coat-A-Count Cortisol assay (Siemens Healthcare Diagnostics, Inc., Los Angeles, USA), a direct double-antibody radioimmunoassay, was used for the determination of salivary cortisol levels. The kit was used according to the manufacturer’s instructions. The analytical sensitivity of the assay was 0.01 μg/dL and the intra-assay coefficient of variation was 3% for samples with a mean concentration of 0.19 ± 0.10 μg/dL and 4% for samples with a mean concentration of 0.24 ± 0.02 μg/dL. The inter-assay coefficient of variation was 12% for samples with a mean concentration of 1.85 ± 0.10 μg/dL and 14% for samples with a mean concentration of 0.24 ± 0.02 μg/dL.

#### Measurement of circadian gene expression level in buccal epithelial cells

The expression levels of circadian rhythm-related genes *PER1* and *ARNTL* were determined by reverse transcription-quantitative polymerase chain reaction (RT-qPCR) and the *PER1/ARNTL* ratio at each sampling time point was used to measure circadian rhythm. Total RNA was isolated from buccal epithelial cells using the RNeasy Micro Kit (Qiagen Inc., Valencia, CA, USA). The final elution of RNA was performed using 20 μL of RNase-free water. To synthesize cDNA, the whole RNA sample was reverse-transcribed in 40 μL reactions using the Sensiscript Reverse Transcription Kit (Qiagen Inc., Valencia, CA, USA) according to the manufacturer’s protocol. Two-microliter aliquots of cDNA were amplified using Taqman PCR reactions in an Applied Biosystems StepOnePlus Real-Time PCR System (ThermoFisher Scientific, Foster City, CA, USA). The primers and Taqman probes used in this experiment are as follows; *PER1* (NM_002616): forward 5′-CTCACACAGCTCCTCCTCAG-3′, reverse 5′-TTTGTGCTCTTGCTGCTCTC-3′, probe 5′FAM-CGGCAAGGACTCAGCCCTGC-3′BHQ1; *ARNTL* (NM_001030272): forward 5′-TGCCTCGTCGCAATTGG-3′, reverse 5′-ACCCTGATTTCCCCGTTCA-3′, probe 5′FAM-CGACTGCATTCTCATGTAGTTCCACAACCA-3′BHQ1.

#### Measurement of actigraphy data

We measured activity, sleep, and light exposure for each participant using the wrist actigraph and the Philips Respironics Actiware software Version 6.0.4 (Philips Respironics, Bend, OR, USA). Participants continuously wore the wrist actigraph on the non-dominant hand and only removed it during times when it might have got wet or damaged. All participants also completed a standardized diary to provide daily records of bedtime, wake-up time, naps, daytime activities, and any periods during which the wrist actigraph was removed. Actigraphy data collected from the wrist actigraph were compared with the self-reported standardized diaries to verify their accuracy.

#### Measurement of sleep parameters

Sleep parameters from NPSG were scored manually according to the standard criteria of the American Academy of Sleep Medicine manual for the scoring of sleep and associated events^73^. Sleep parameters such as total sleep time (TST); sleep efficiency (SE); wake after sleep onset (WASO); sleep latency (SL); stages N1, N2, N3, and R, stage R latency; REM density; apnea hypopnea index (AHI); respiratory effort-related arousal index (RERA index); respiratory disturbance index (RDI; AHI + RERA index); percentage of supine position (Supine); periodic limb movement during sleep index (PLMS index); limb movement index (LM index); total arousal time (TA); and spontaneous arousal time (SA) were extracted from the scored results of each NPSG session. All sleep stages (N1, N2, N3, and R) were converted to percentages before analysis to adjust for the difference in TST of each subject on each night. We encountered technical problems, which led to incomplete data collection during the NPSG session for one case in the high MDQ score group and for three cases in the low MDQ score group. In total, NPSG data from 21 subjects (13 subjects in the high MDQ score group and 8 subjects in the low MDQ score group) were analyzed.

### Data Analysis

Salivary cortisol concentrations and relative *PER1/ARNTL* gene expression levels at 8:00 h, 12:00 h, 16:00 h, 20:00 h, and 24:00 h from the last two consecutive days of each of the two different ALAN schedules were fitted with sine curves for sine regression analysis. Variables such as period, amplitude, acrophase, and mesor for each of the molecular circadian rhythms were then extracted for analysis. Sine regression analysis was performed using SigmaPlot software Version 10.0 (Systat Software, Inc., San Jose, CA, USA). The period, robustness, mesor, amplitude, and acrophase variables for behavioral rhythm were calculated from the physical activity data and analyzed using Cosinor software (Circadian Rhythm Laboratory of Boise State University, Boise, ID, USA).

In order to determine the impact of bright ALAN exposure before bedtime on all of the subjects, the circadian rhythm variables of salivary cortisol concentration, relative *PER1/ARNTL* gene expression levels, and behavioral rhythm and sleep parameters for each night were analyzed using paired *t*-tests or Wilcoxon signed-rank tests. The appropriate statistical test was chosen based on the results of normality tests performed for each of the variables using both the Kolmogorov-Smirnov and the Shapiro-Wilk methods.

To find differential effects of bright ALAN exposure before bedtime on the different groups on different nights, a repeated measures analysis of variance (ANOVA) was performed for each variable.

## Additional Information

**How to cite this article**: Cho, C.-H. *et al.* Molecular circadian rhythm shift due to bright light exposure before bedtime is related to subthreshold bipolarity. *Sci. Rep.* **6**, 31846; doi: 10.1038/srep31846 (2016).

## Supplementary Material

Supplementary Information

## Figures and Tables

**Table 1:** Results of paired *t*-tests or Wilcoxon signed-rank tests on the circadian rhythm variables of salivary cortisol concentration and relative *PER1/ARNTL* gene expression levels between Night 1 and Night 2.

Type of sample	Variables	Night 1	Night 2	t or Z	df	p-value(two tailed)
		Mean ± SD	Mean ± SD			
Cortisol concentration (n = 25)	Period (hr)	24.813 ± 0.777	25.175 ± 0.985	−1.523	24	0.141
	Amplitude (μg/dL)	0.217 ± 0.033	0.205 ± 0.064	0.807	24	0.428
	Acrophase (time)	9.935 ± 0.964	11.646 ± 1.936	−3.927	24	**0.001**^**†**^
	Mesor (μg/dL)	0.272 ± 0.036	0.307 ± 0.048	−2.494	24	**0.020**^**†**^
Relative *PER1/ARNTL* gene expression (n = 25)	Period (hr)^*^	24.237 ± 0.747	24.215 ± 0.880	0.040	—	0.968
	Amplitude	28.186 ± 3.487	22.103 ± 4.993	6.555	24	**<0.001**^**‡**^
	Acrophase (time)	15.886 ± 0.592	18.504 ± 0.965	−11.146	24	**<0.001**^**‡**^
	Mesor	25.919 ± 3.197	22.983 ± 4.698	2.638	24	**0.014**^**†**^

^†^*p*-value < 0.05.

^‡^*p*-value < 0.001.

^*^Wilcoxon signed-rank test.

Night 1, exposure to artificial light of 150-lux intensity at night.

Night 2, exposure to artificial light of 1,000-lux intensity at night.

Amplitude and Mesor of Relative *PER1/ARNTL* gene expression indicate the relative *PER1/ARNTL* gene expression ratio.

All variables except those tested using the Wilcoxon signed-rank test were analyzed by a paired *t*-test.

hr, hour; time, time of the day; TST, total sleep time; min, minutes; n, number of subjects; SD, standard deviation; and df, degrees of freedom.

**Table 2:** Results of a repeated measures ANOVA of the circadian rhythm variables of salivary cortisol concentration and relative *PER1/ARNTL* gene expression levels between Nights 1 and 2 and high and low mood disorder questionnaire (MDQ) score groups.

Type of sample	Variables	Nights	High MDQ score group (n = 14 or 13)	Low MDQ score group (n = 11 or 8)	Source	F	*p*
			Mean ± SD	Mean ± SD			
Cortisol concentration (n = 25)	Period (hr)	Night 1	24.847 ± 0.838	24.769 ± 0.729	Nights	2.518	0.126
		Night 2	25.051 ± 0.913	25.333 ± 1.093	Group	0.142	0.710
					Night*Group	0.554	0.464
	Amplitude (μg/dL)	Night 1	0.223 ± 0.031	0.209 ± 0.036	Nights	0.809	0.378
		Night 2	0.222 ± 0.064	0.182 ± 0.06	Group	4.248	0.051
					Night*Group	0.033	0.400
	Acrophase (time)	Night 1	9.866 ± 1.114	10.021 ± 0.775	Nights	16.956	**<0.001**^**‡**^
		Night 2	12.562 ± 1.668	10.481 ± 1.647	Group	5.995	**0.022**^**†**^
					Night*Group	8.528	**0.008**^**†**^
	Mesor (μg/dL)	Night 1	0.265 ± 0.035	0.283 ± 0.036	Nights	5.678	**0.026**^**†**^
		Night 2	0.321 ± 0.039	0.288 ± 0.054	Group	0.531	0.473
					Night*Group	3.792	0.064
Relative *PER1/ARNTL* gene expression (n = 25)	Period (hr)	Night 1	24.225 ± 0.724	24.252 ± 0.81	Nights	0.01	0.920
		Night 2	24.226 ± 0.917	24.2 ± 0.875	Group	<0.001	0.999
					Night*Group	0.011	0.918
	Amplitude	Night 1	27.383 ± 3.182	29.209 ± 3.737	Nights	44.774	**<0.001**^**‡**^
		Night 2	22.243 ± 5.848	21.925 ± 3.913	Group	0.258	0.617
					Night*Group	1.335	0.260
	Acrophase (time)	Night 1	16.118 ± 0.549	15.591 ± 0.529	Nights	122.1	**<0.001**^**‡**^
		Night 2	18.58 ± 0.906	18.408 ± 1.072	Group	2.728	0.112
					Night*Group	0.549	0.466
	Mesor	Night 1	25.799 ± 3.38	26.072 ± 3.105	Nights	7.338	**0.013**^**†**^
		Night 2	23.722 ± 5.321	22.044 ± 3.798	Group	0.352	0.559
					Night*Group	0.750	0.396

^†^*p*-value < 0.05.

^‡^*p*-value < 0.001.

Night 1, exposure to artificial light of 150-lux intensity at night.

Night 2, exposure to artificial light of 1000-lux intensity at night.

Amplitude and Mesor of Relative *PER1/ARNTL* gene expression indicate the relative ratio of *PER1/ARNTL* gene expression.

hr, hour; time, time of the day; min, minutes; n, number of subjects; and SD, standard deviation.

**Table 3:** Results of paired *t*-tests or Wilcoxon signed-rank tests of the behavioral rhythm and sleep parameters between Night 1 and Night 2.

Type of sample	Variables	Night 1	Night 2	t or Z	df	p-value (two tailed)
		Mean ± SD	Mean ± SD			
Behavioral rhythm (n = 25)	Period (hr)	24.064 ± 0.617	23.900 ± 0.585	1.071	24	0.295
	Amplitude	168.331 ± 53.029	184.816 ± 53.643	−1.085	24	0.289
	Acrophase (time)	15.649 ± 1.714	15.332 ± 1.436	0.662	24	0.515
	Mesor	243.126 ± 71.803	244.961 ± 59.853	−0.098	24	0.923
	Robustness (%)	24.008 ± 7.854	23.476 ± 5.277	0.301	24	0.766
Sleep parameters (n = 21)	TST (min)^*^	384.380 ± 18.202	385.548 ± 16.381	−0.818	—	0.413
	SE (%)	97.433 ± 1.580	97.405 ± 1.775	0.099	20	0.922
	WASO (min)^*^	6.024 ± 5.278	5.74 ± 5.449	−1.252	—	0.211
	SL (min)	4.000 ± 2.617	4.520 ± 3.120	−0.732	20	0.473
	Stage N1 (%)	12.262 ± 4.923	13.395 ± 6.688	−1.213	20	0.239
	Stage N2 (%)	45.267 ± 5.791	44.467 ± 5.865	0.535	20	0.598
	Stage N3 (%)	18.638 ± 7.386	17.557 ± 8.642	0.994	20	0.332
	Stage R (%)	23.848 ± 4.777	24.571 ± 3.761	−0.818	20	0.423
	Stage R latency (min)^*^	71.430 ± 42.432	71.762 ± 40.937	−0.709	—	0.478
	RDI^*^	4.067 ± 2.923	4.090 ± 3.470	−0.348	—	0.728
	AHI	2.433 ± 0.382	2.400 ± 2.399	0.117	20	0.908
	RERAI	1.633 ± 1.451	1.720 ± 1.591	−0.633	20	0.534
	PLMI^*^	2.150 ± 5.899	2.929 ± 8.166	−0.691	—	0.490
	LMI^*^	5.676 ± 6.419	6.933 ± 9,168	−0.869	—	0.385
	TA (min)	12.952 ± 4.249	13.410 ± 5.311	−0.718	20	0.481
	SA (min)	8.052 ± 3.800	8.424 ± 4.537	−0.688	20	0.499
	Supine position (%)^*^	77.790 ± 18.453	80.252 ± 20.704	−0.262	—	0.793

^†^*p*-value < 0.05.

^*^Wilcoxon signed-rank test.

Night 1, exposure to artificial light of 150-lux intensity at night.

Night 2, exposure to artificial light of 1,000-lux intensity at night.

All variables except those tested using the Wilcoxon signed-rank test were analyzed by a paired *t*-test.

hr, hour; time, time of the day; TST, total sleep time; SE, sleep efficiency; WASO, wake time after sleep onset; SL, sleep latency; AHI, apnea + hypopnea index; RDI, respiratory disturbance index; RERAI, respiratory effort-related arousal index; PLMI, periodic limb movement during sleep index; LMI, limb movement index; TA, total arousal; SA, spontaneous arousal; min, minutes; n, number of subjects; SD, standard deviation; and df, degrees of freedom.

**Table 4:** Results of a repeated measures ANOVA of the behavioral rhythm and sleep parameters between Nights 1 and 2 and high and low mood disorder questionnaire (MDQ) score groups.

Type of sample	Variables	Nights	High MDQ score group (n = 14 or 13)	Low MDQ score group (n = 11 or 8)	Source	F	*p*
			Mean ± SD	Mean ± SD			
Behavioral rhythm (n = 25)	Period (hr)	Night 1	23.914 ± 0.728	24.255 ± 0.391	Nights	2.375	0.137
(n = 25)		Night 2	24.086 ± 0.429	23.664 ± 0.687	Group	0.046	0.832
					Night*Group	7.844	**0.010**^**†**^
	Amplitude	Night 1	157.870 ± 37.402	181.644 ± 67.692	Nights	0.839	0.369
		Night 2	196.098 ± 47.618	170.456 ± 59.610	Group	0.004	0.952
					Night*Group	2.804	0.108
	Acrophase (time)	Night 1	15.339 ± 1.695	16.045 ± 1.736	Nights	0.518	0.479
		Night 2	15.279 ± 1.359	15.399 ± 1.593	Group	0.987	0.331
					Night*Group	0.358	0.555
	Mesor	Night 1	218.217 ± 74.838	274.828 ± 55.845	Nights	0.013	0.911
		Night 2	248.568 ± 66.164	240.369 ± 53.528	Group	1.713	0.204
					Night*Group	3.212	0.086
	Robustness (%)	Night 1	24.921 ± 9.231	22.845 ± 5.884	Nights	0.047	0.830
		Night 2	23.364 ± 4.939	23.618 ± 5.922	Group	0.196	0.662
					Night*Group	0.417	0.525
Sleep parameters	TST (min)	Night 1	387.12 ± 19.777	379.94 ± 15.481	Nights	0.658	0.427
(n = 21)		Night 2	387.038 ± 17.578	383.125 ± 15.038	Group	0.528	0.476
					Night*Group	0.725	0.405
	SE (%)	Night 1	97.246 ± 1.624	97.738 ± 1.561	Nights	0.006	0.940
		Night 2	97.200 ± 2.010	97.738 ± 1.369	Group	0.536	0.473
					Night*Group	0.006	0.940
	WASO (min)	Night 1	6.462 ± 4.850	5.313 ± 6.193	Nights	0.255	0.619
		Night 2	6.500 ± 5.583	4.500 ± 5.345	Group	0.460	0.506
					Night*Group	0.309	0.585
	SL (min)	Night 1	4.308 ± 2.905	3.500 ± 2.155	Nights	0.643	0.432
		Night 2	4.58 ± 2.907	4.44 ± 3.649	Group	0.190	0.668
					Night*Group	0.197	0.662
	Stage N1 (%)	Night 1	13.046 ± 5.067	10.988 ± 4.716	Nights	0.729	0.404
		Night 2	15.262 ± 7.280	10.363 ± 4.469	Group	2.116	0.162
					Night*Group	2.325	0.144
	Stage N2 (%)	Night 1	45.708 ± 6.552	44.550 ± 4.612	Nights	0.579	0.456
		Night 2	46.092 ± 6.024	41.825 ± 4.815	Group	1.696	0.208
					Night*Group	1.022	0.325
	Stage N3 (%)	Night 1	17.738 ± 8.495	20.100 ± 5.310	Nights	0.283	0.601
		Night 2	14.946 ± 9.784	21.800 ± 4.009	Group	1.879	0.186
					Night*Group	4.785	**0.041**^**†**^
	Stage R (%)	Night 1	23.508 ± 5.740	24.400 ± 2.852	Nights	0.946	0.343
		Night 2	23.685 ± 3.926	26.013 ± 3.186	Group	0.888	0.358
					Night*Group	0.609	0.445
	Stage R latency (min)	Night 1	77.88 ± 45.513	60.94 ± 37.282	Nights	0.017	0.897
		Night 2	76.346 ± 46.806	64.313 ± 30.495	Group	0.679	0.420
					Night*Group	0.124	0.729
	RDI	Night 1	4.646 ± 3.096	3.125 ± 2.518	Nights	0.011	0.918
		Night 2	4.62 ± 3.655	3.23 ± 3.179	Group	1.099	0.308
					Night*Group	0.028	0.869
	AHI	Night 1	2.823 ± 1.836	1.800 ± 1.489	Nights	0.001	0.971
		Night 2	2.62 ± 2.690	2.03 ± 1.946	Group	0.838	0.371
					Night*Group	0.391	0.539
	RERAI	Night 1	2.07 ± 2.53	1.51 ± 1.34	Nights	2.246	0.149
		Night 2	1.63 ± 1.96	1.28 ± 1.17	Group	1.027	0.324
					Night*Group	0.198	0.661
	PLMI	Night 1	3.28 ± 7.356	0.33 ± 0.709	Nights	0.662	0.426
		Night 2	4.223 ± 10.2807	0.825 ± 1.086	Group	1.065	0.315
					Night*Group	0.063	0.805
	LMI	Night 1	6.892 ± 7.894	3.700 ± 1.922	Nights	1.505	0.235
		Night 2	8.169 ± 11.440	4.925 ± 2.895	Group	0.883	0.359
					Night*Group	0.001	0.980
	TA (min)	Night 1	13.638 ± 4.409	11.838 ± 3.996	Nights	0.054	0.820
		Night 2	15.115 ± 5.842	10.638 ± 2.811	Group	2.498	0.130
					Night*Group	5.000	**0.038**^**†**^
	SA (min)	Night 1	8.046 ± 3.649	8.063 ± 4.294	Nights	0.036	0.852
		Night 2	9.300 ± 5.302	7.000 ± 2.622	Group	0.392	0.539
					Night*Group	5.266	0.033
	Supine position (%)	Night 1	76.823 ± 21.330	79.363 ± 13.726	Nights	0.882	0.359
		Night 2	78.177 ± 21.308	83.625 ± 20.624	Group	0.222	0.643
					Night*Group	0.237	0.632

^†^*p*-value < 0.05.

Night 1, exposure to artificial light of 150-lux intensity at night.

Night 2, exposure to artificial light of 1000-lux intensity at night.

The numbers of subjects in the high MDQ score group (n = 14) and the low MDQ score group (n = 11) are for the variables of behavioral rhythm. However, sleep parameters have different numbers of subjects in each group: high MDQ score group (n = 13) and low MDQ score group (n = 8).

hr, hour; time, time of the day; TST, total sleep time; SE, sleep efficiency; WASO, wake time after sleep onset; SL, sleep latency; AHI, apnea + hypopnea index; RDI, respiratory disturbance index; RERAI, respiratory effort-related arousal index; PLMI, periodic limb movement during sleep index; LMI, limb movement index; TA, total arousal; SA, spontaneous arousal; min, minutes; n, number of subjects; and SD, standard deviation.
