# Supplementary material for: Molecular circadian rhythm shift due to bright light exposure before bedtime is related to subthreshold bipolarity
Source: Sci Rep. 2016 Aug 22;6:31846. doi: 10.1038/srep31846 (PMC4992827; doi:10.1038/srep31846)

Supplementary Information

Molecular circadian rhythm shift due to bright light exposure before bedtime is related to subthreshold bipolarity

Chul-Hyun Cho[1,2,†], Joung-Ho Moon[3,†], Ho-Kyoung Yoon[1,2], Seung-Gul Kang[4], Dongho Geum[3], Gi-Hoon Son[5], Jong-Min Lim[6], Leen Kim[1, 2], Eun-Il Lee[7], Heon-Jeong Lee[1, 2, 3]*

[1] *Department of Psychiatry, Korea University College of Medicine, Seoul, South Korea*

[2] *Sleep-Wake Disorders Center, Korea University Anam Hospital, Seoul, South Korea*

[3] *Department of Biomedical Science, Korea University College of Medicine, Seoul, South Korea*

[4] *Department of Psychiatry, Gachon University School of Medicine, Incheon, South Korea*

[5] *Department of Legal Medicine, Korea University College of Medicine, Seoul, South Korea*

[6] *Department of Lighting Environment Research, Korea Institute of Lighting Technology, Seoul, South Korea*

[7] *Department of Preventive Medicine, Korea University College of Medicine, Seoul, South Korea*

[†]These authors contributed equally to this work.

*Corresponding author at: Department of Psychiatry, Anam Hospital, Korea University College of Medicine, Anam-dong 5-ga, Seongbuk-gu, Seoul 136-705, South Korea.

Tel.: +82-2-920-5815; fax: +82-2-929-7679.

E-mail address: leehjeong@korea.ac.kr (H.-J. Lee).

Supplemental Figure S1. Circadian gene expression in buccal epithelial cells of five healthy people (independent from the present main study) who had been confirmed to show regular circadian rhythms: preliminary research. Five circadian genes (*ARNTL, PER1*, *PER2*, *PER3*, and *NR1D1*) extracted from buccal epithelial cells of five healthy people (independent from the present main study) who had been confirmed to show regular circadian rhythms by actigraphy and salivary cortisol concentration were tested for gene expression. Although distinct circadian rhythms of gene expressions were not shown when each gene was observed alone (A), we observed more relevant circadian rhythms of *ARNTL* and *PER1* with inverse in phase to each other. We found the ratio of *PER1*/*ARNTL* gene expression is a reliable method for measuring circadian rhythms of peripheral circadian gene expression (B).

A.

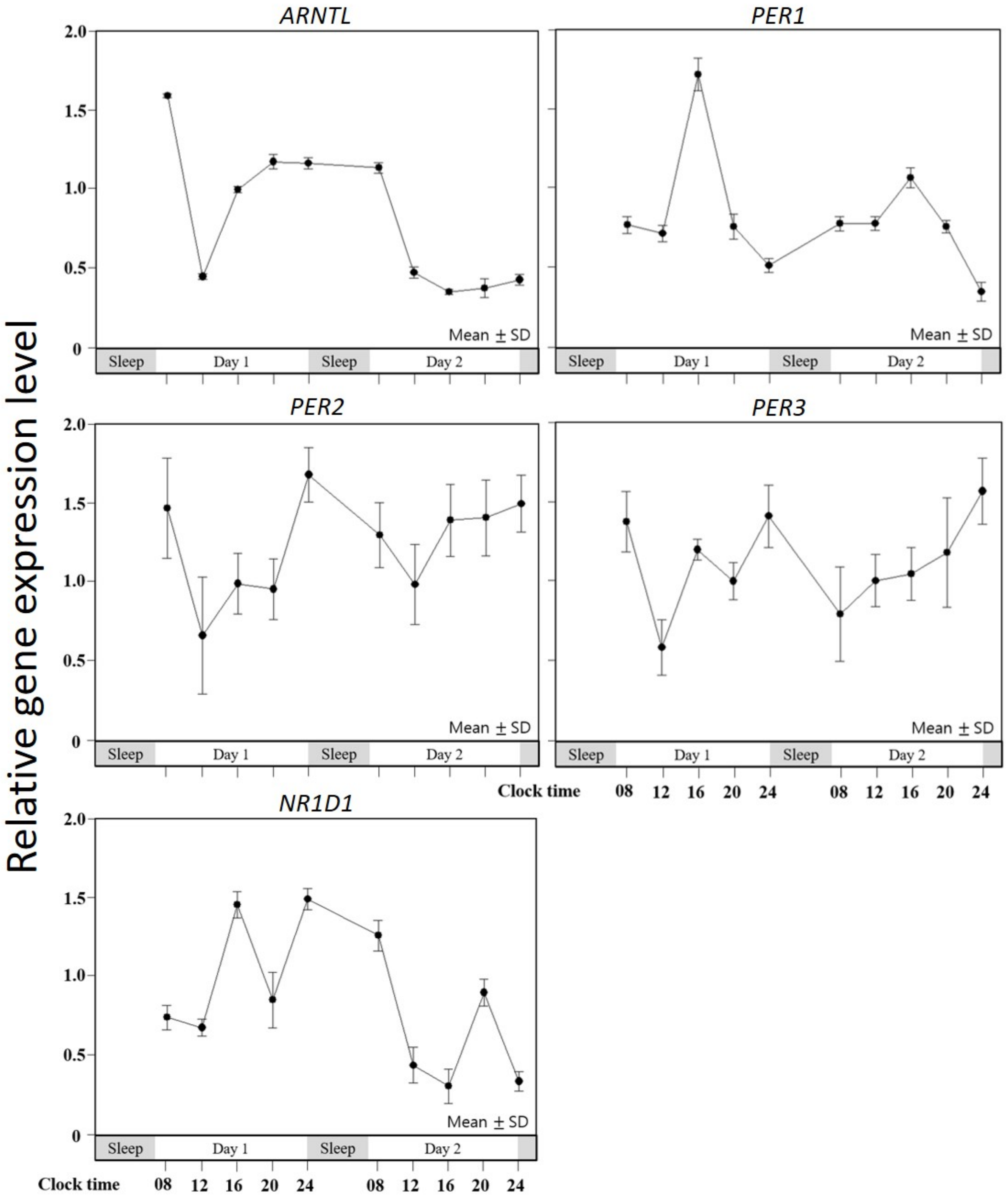
ARNTL
PER1
PER2
PER3
NR1D1
Relative gene expression level
Mean ± SD
Sleep
Day 1
Day 2
Clock time
08
12
16
20
24
2.0
1.5
1.0
0.5
0

B.

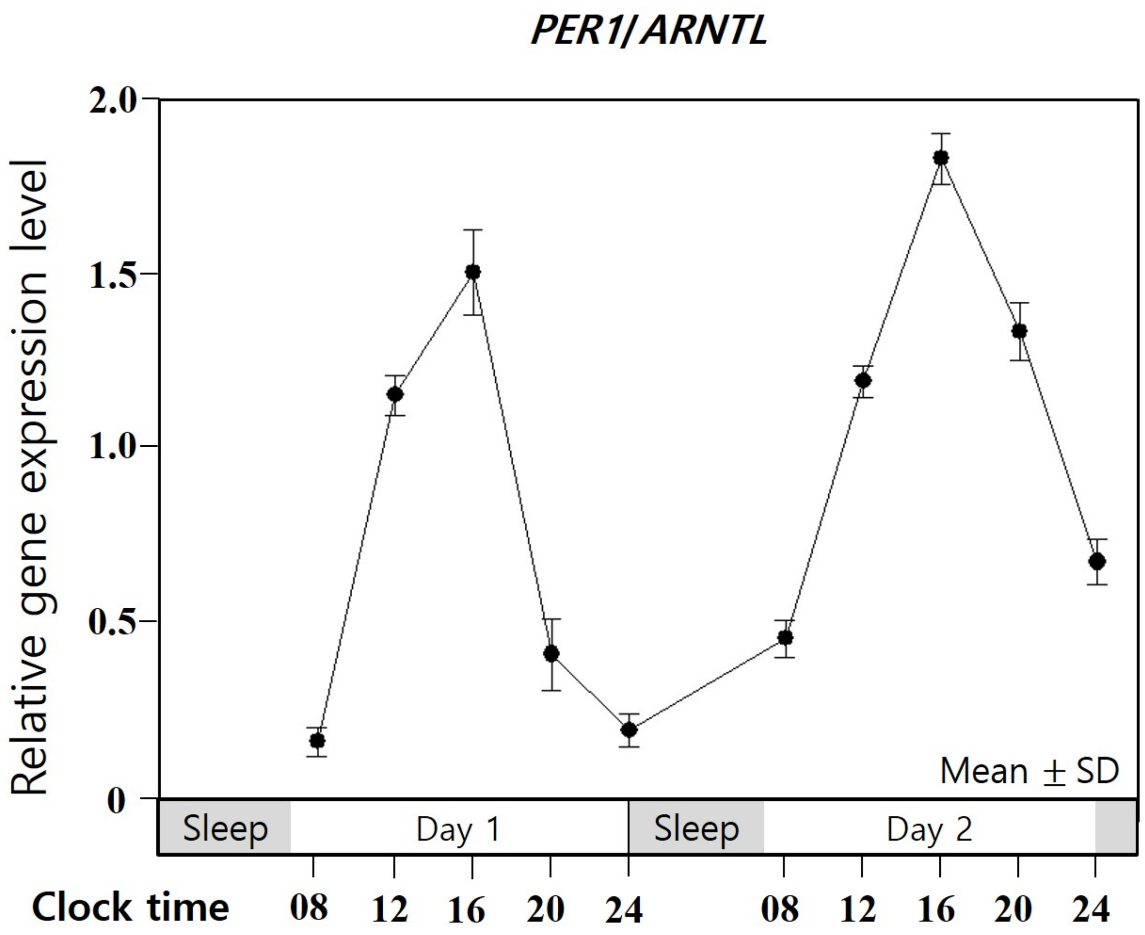

PER1/ARNTL
2.0
1.5
1.0
0.5
0
Relative gene expression level
Mean ± SD
Sleep
Day 1
Sleep
Day 2
Clock time
08
12
16
20
24
08
12
16
20
24

Supplemental Figure S2. Relative gene expression level of *ARNTL*, *PER1, and PER1/ARNTL* in buccal epithelial cells of one representative subject in low mood disorder questionnaire (MDQ) score group under 150 lux condition.

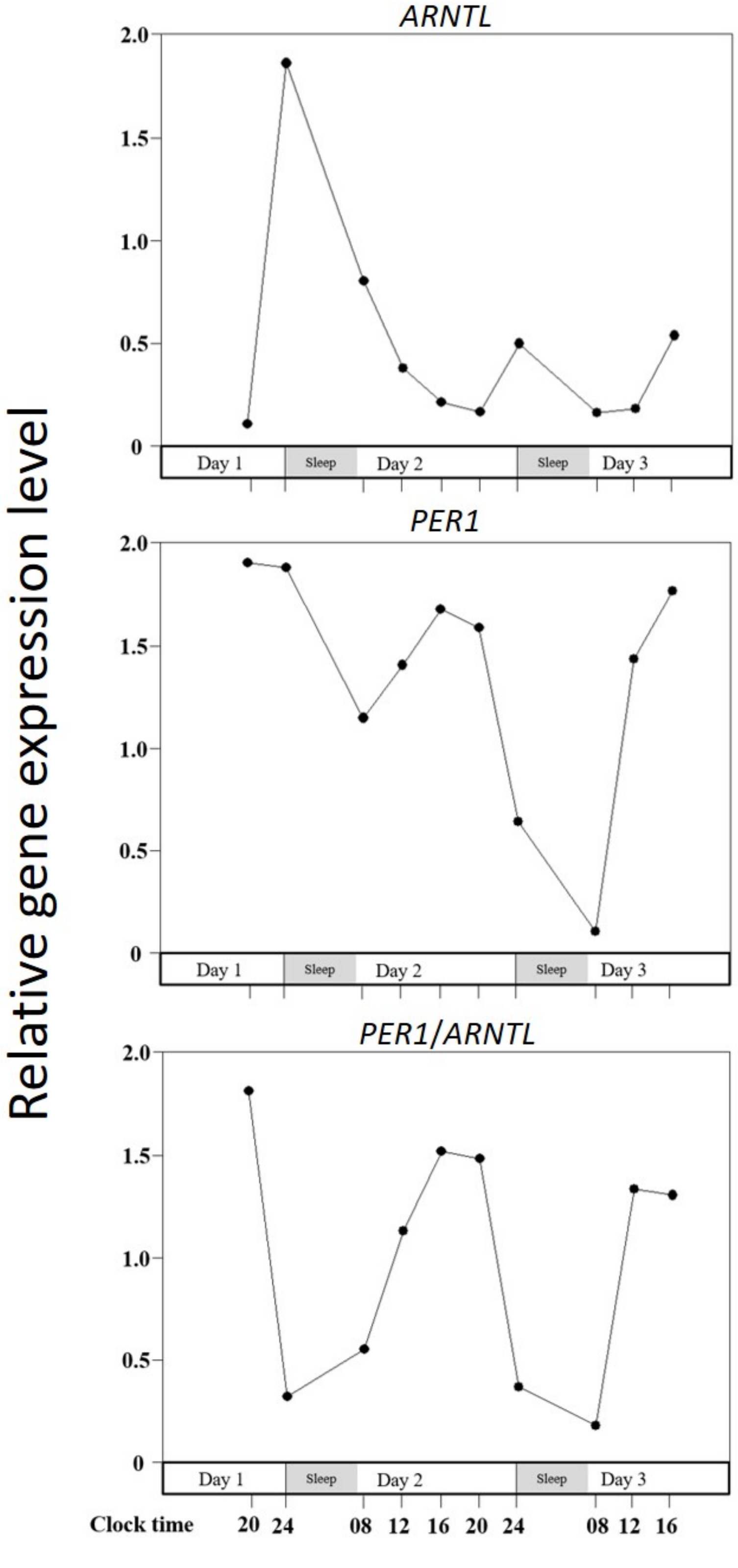

Supplement: Supplementary Information [file srep31846-s1.pdf]
